# Supplementary material for: Hippocampal microcircuits constrain the generation of epileptiform activity
Source: bioRxiv. 2026 Jun 30:2026.06.25.734494. Preprint. [Version 1] doi: 10.64898/2026.06.25.734494 (PMC13345148; doi:10.64898/2026.06.25.734494)
Supplement: Supplement 1 [file NIHPP2026.06.25.734494v1-supplement-1.pdf]

bioRxiv preprint doi: https://doi.org/10.64898/2026.06.25.734494; this version posted June 30, 2026. The copyright holder for this preprint (which was not certified by peer review) is the author/funder, who has granted bioRxiv a license to display the preprint in perpetuity. It is made available under a CC-BY 4.0 International license.

Supplemental Information

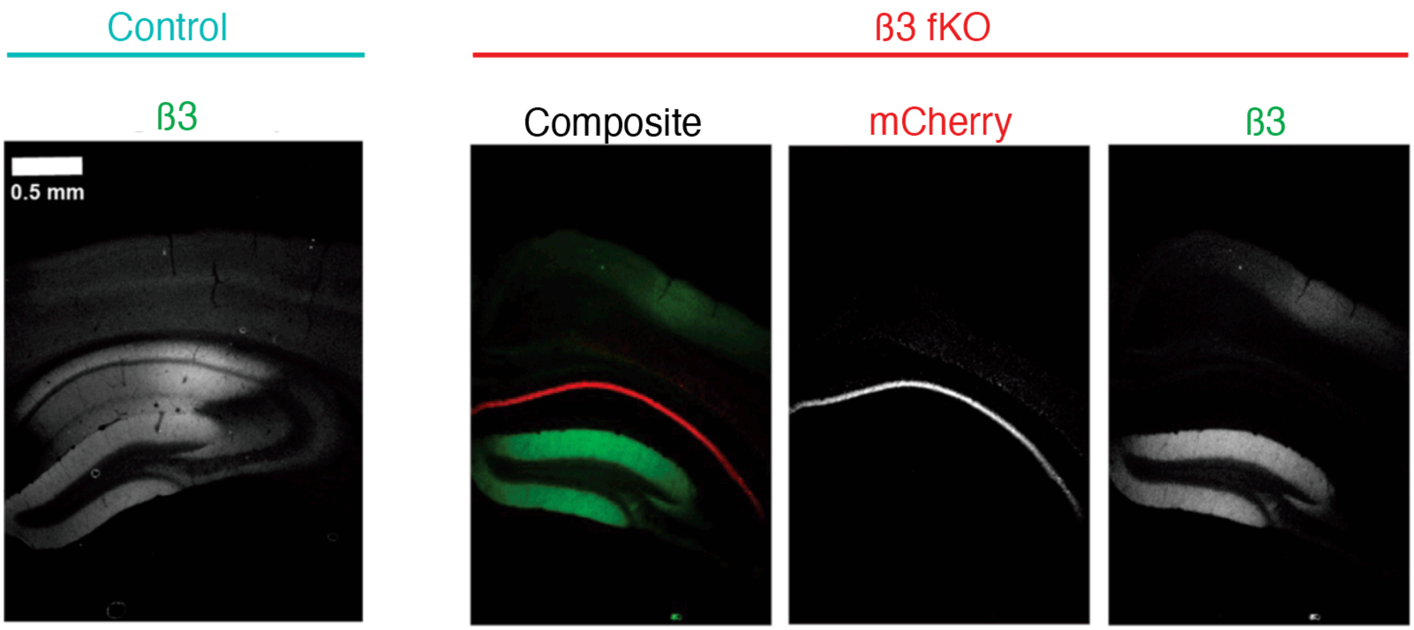

Figure S1: Focal loss of β3 in *Gabrb3fl/fl* mice following AAV-CaMKII-Cre-mCherry injection
Compared to control mice with abundant β3 expression, β3 fKO mice display an absence of β3 expression. Notably, β3 expression is spared in the dentate gyrus, confirming the specificity of the knockout strategy.

bioRxiv preprint doi: https://doi.org/10.64898/2026.06.25.734494; this version posted June 30, 2026. The copyright holder for this preprint (which was not certified by peer review) is the author/funder, who has granted bioRxiv a license to display the preprint in perpetuity. It is made available under a CC-BY 4.0 International license.

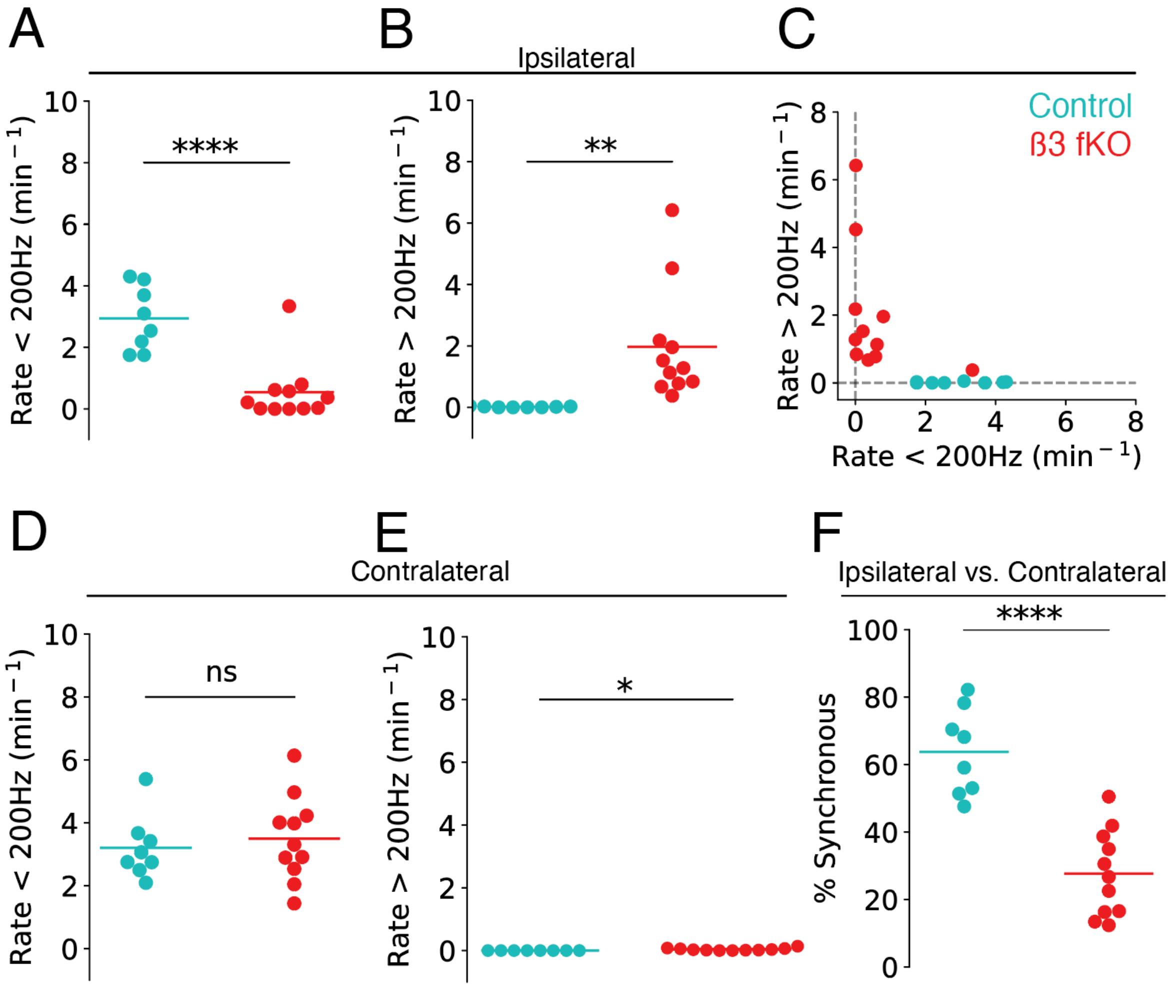

Figure S2: Pathological HFOs localize to the ipsilateral CA1 in β3 fKO mice
(A) β3 fKO mice have fewer physiological ripples (<200Hz) than control mice ipsilateral to the AAV injection site. $t_{17} = 5.16$, $p = 0.0000791$.
(B) β3 fKO mice have more pathological HFOs (<200Hz) than control mice ipsilateral to the AAV injection site. $t_{17} = 2.95$, $p = 0.00902$.
(C) Data from A and B replotted. An exponential function better fits the data than a linear function (r=0.72 vs. r=-0.60, respectively), suggesting that SPW-Rs and HFOs tend to not co-occur in the same region.
(D) No differences were observed in the rate of contralateral physiological ripples. $t_{17} = 0.51$, $p = 0.614$.
(E) β3 fKO mice have more pathological HFOs (<200Hz) than control mice on the contralateral hemisphere, but the average event rate is very low (0.04 ± 0.012 events per minute in β3 fKO mice versus 0.0 in control mice). $t_{17} = 2.64$, $p = 0.0171$.
(F) Percent of events, inclusive of physiological ripples (<200Hz) and pathological (>200Hz) HFOs, that are coincident with a contralaterally-detected SPW-R. $t_{17} = 6.07$, $p = 0.0000126$.
*p<0.05, p<0.01, ****p<0.0001

bioRxiv preprint doi: https://doi.org/10.64898/2026.06.25.734494; this version posted June 30, 2026. The copyright holder for this preprint (which was not certified by peer review) is the author/funder, who has granted bioRxiv a license to display the preprint in perpetuity. It is made available under a CC-BY 4.0 International license.

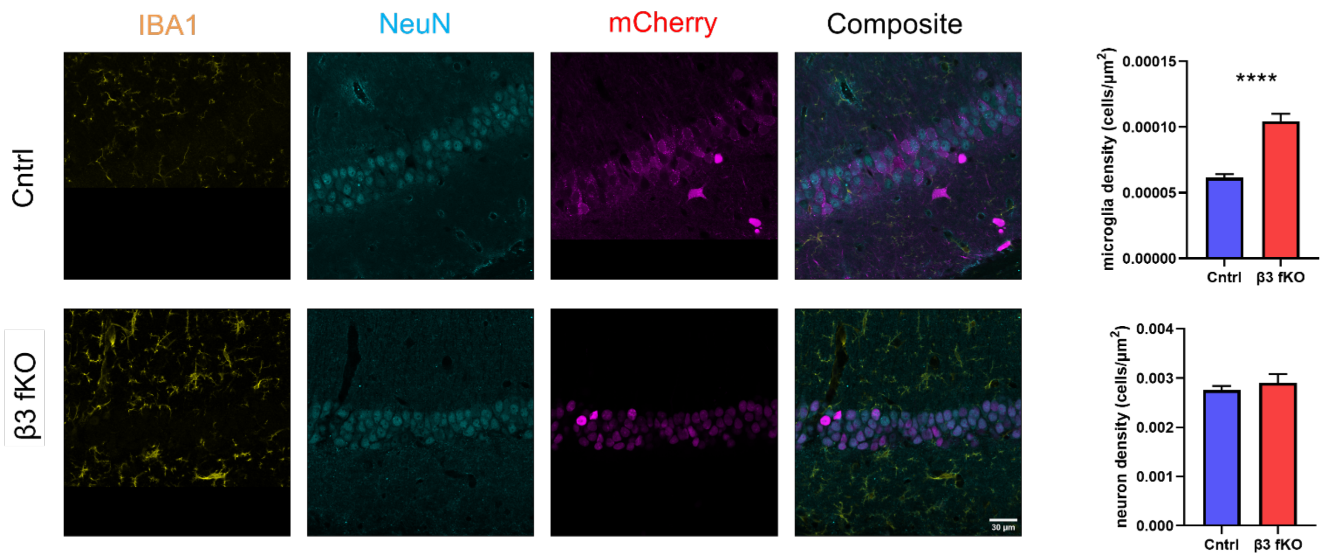

Figure S3: Histological characterization of inflammation and cell death in β3 fKO mice
Increased inflammation ($p<0.0001$) but no cell death ($p=0.9742$) observed in CA1 tissue from β3 focal knockout mice (n=12 sections/4mice control, 10 sections/4mice β3 focal knockout).
